# Clinical Application of Ventricular Intracranial Pressure Monitoring in the Treatment of Ruptured Anterior Circulation Aneurysms: A Retrospective Study

**DOI:** 10.7759/cureus.79138

**Published:** 2025-02-17

**Authors:** Tengda Chen, Xieli Guo, Mingfa Cai

**Affiliations:** 1 Neurological Surgery, Jinjiang Municipal Hospital (Shanghai Sixth People's Hospital Fujian), Quanzhou, CHN

**Keywords:** anterior circulation aneurysm, icp monitoring, neurological recovery, postoperative complications, surgical clipping

## Abstract

Objective: To evaluate the clinical effectiveness of ventricular intracranial pressure (ICP) monitoring in the treatment of ruptured anterior circulation aneurysms and to explore its impact on intracranial pressure control, postoperative complications, length of hospital stay, and neurological recovery.

Methods: A retrospective analysis was conducted on 67 patients who underwent surgical clipping for ruptured anterior circulation aneurysms at Jinjiang Hospital, Fujian Province, between January 2021 and June 2024. The patients were divided into an experimental group (32 patients) and a control group (35 patients). Clinical characteristics, operation time, intraoperative blood loss, postoperative changes in ICP, postoperative complications, length of hospital stay, and neurological recovery were compared between the two groups.

Results: No significant differences were found between the experimental and control groups in terms of clinical characteristics, operation time, or intraoperative blood loss (P > 0.05). However, the experimental group showed superior outcomes compared to the control group in postoperative ICP changes, simplified early brain edema score (SEBES) at 72 hours, length of hospital stay, and Glasgow Outcome Scale (GOS) scores at discharge, with statistically significant differences (P < 0.05).

Conclusion: Ventricular ICP monitoring during the surgical clipping of ruptured anterior circulation aneurysms is an effective method for controlling intracranial pressure, reducing postoperative complications, shortening the length of hospital stay, and promoting neurological recovery. This approach offers a safer and more efficient monitoring solution with promising clinical applicability.

## Introduction

Intracranial aneurysms are protrusions formed by abnormal bulging of the arterial wall, with anterior circulation aneurysms accounting for approximately 85% of all intracranial aneurysms [1,2]. Aneurysmal subarachnoid hemorrhage (aSAH) is a serious disease that threatens the health of the Chinese population, with a mortality rate as high as 50% [3]. For survivors of aSAH, acute brain injury often leads to severe physical or cognitive impairments, significantly affecting their workability and quality of life [4]. Therefore, improving the treatment of aSAH patients and enhancing their prognosis has become a critical issue that urgently needs to be addressed.

After the rupture of an aneurysm, a large amount of blood enters the subarachnoid space, leading to intracranial pressure (ICP). Elevated ICP can severely impair brain function, triggering a series of irreversible neurological deficits. Additionally, factors such as the mass effect caused by bleeding and disturbances in cerebrospinal fluid circulation further exacerbate the pathophysiological deterioration caused by increased ICP. A systematic review and meta-analysis showed that the incidence of ICP exceeding 20 mmHg in aSAH patients is as high as 70.69%, with high-grade aSAH patients being more prone to ICP elevation [5]. In 2021, a multicenter prospective observational cohort study published in Lancet Neurology indicated that brain injury patients, including those with SAH, who received ICP monitoring-guided treatment had a lower six-month mortality rate and better neurological outcomes [6]. ICP monitoring provides real-time, dynamic, and accurate data on intracranial pressure, allowing for precise ICP control and timely adjustments to treatment strategies. Current guidelines clearly recommend the use of ICP monitoring in patients with traumatic brain injury, particularly those with severe brain injuries requiring intensive neurological care [7]. However, the management of ICP in SAH patients is primarily based on studies of traumatic brain injury, with a lack of high-level randomized controlled trial (RCT) evidence directly targeting SAH patients. Therefore, the specific application of ICP monitoring in aSAH patients remains controversial, and there are currently no relevant authoritative guidelines.

Given the potential importance of ICP monitoring in the management of aSAH and its lack of sufficient high-level evidence-based medical support in clinical practice, this study will retrospectively analyze 67 patients who underwent clipping surgery for anterior circulation ruptured aneurysms with concurrent ICP monitoring at our hospital. The aim is to explore the effectiveness of ventricular ICP monitoring in improving neurological recovery, shortening hospital stays, and reducing complications in these patients.

## Materials and methods

General information

This study was approved by the Ethics Committee of Jinjiang Hospital (Fujian Branch of Shanghai Sixth People's Hospital) and was conducted in accordance with the Declaration of Helsinki. As a retrospective study, the ethics committee waived the requirement for informed consent, in compliance with Chinese laws and institutional regulations. A total of 67 patients who underwent clipping surgery for anterior circulation ruptured aneurysms at Jinjiang Hospital (Fujian Branch of Shanghai Sixth People's Hospital) from January 2021 to June 2024 were selected. All patients were diagnosed with anterior circulation aneurysm rupture and subarachnoid hemorrhage based on imaging examinations. The patients included 29 males and 38 females, with ages ranging from 32 to 69 years.

The inclusion criteria for this study were as follows: patients aged between 18 and 75 years; diagnosed with anterior circulation ruptured aneurysms through computed tomography angiography (CTA) or digital subtraction angiography (DSA); ruptured aneurysm leading to subarachnoid hemorrhage without severe preoperative complications, such as brain herniation or large infarction; underwent aneurysm clipping surgery and ICP monitoring under a microscope. The exclusion criteria included: ruptured posterior circulation aneurysms; contraindications to lumbar puncture; irreversible brain injury prior to surgery, such as brain herniation or large infarction; inability to complete follow-up or missing postoperative follow-up data.

Clinical and imaging characteristics

The initial symptoms of the patients were headache and meningeal irritation, accompanied by varying degrees of consciousness disturbance, with the time from onset to surgery as short as three hours. Preoperative Hunt-Hess grading showed that 54 cases were graded I-III and 13 cases were graded IV-V. All patients underwent CT scans, which revealed subarachnoid hemorrhage and were subsequently diagnosed through CTA or DSA. The aneurysms were distributed as follows: 27 cases of anterior communicating artery aneurysm, 21 cases of middle cerebral artery aneurysm, 15 cases of posterior communicating artery aneurysm, two cases of anterior cerebral artery aneurysm, one case of internal carotid artery petrous segment aneurysm, and one case of choroidal artery aneurysm. The aneurysm size distribution was 23 small, 40 medium, and four large.

Study design

Patients were divided into two groups for comparison based on different treatment protocols: the experimental group comprised patients who underwent aneurysm clipping surgery with ventricular ICP monitoring through the same incision; the control group was patients who underwent lumbar cistern drainage before surgery and traditional aneurysm clipping, along with parenchymal ICP monitoring.

Surgical protocol

All aneurysm clipping surgeries are performed by an experienced neurosurgical team, following a standardized surgical procedure. The specific steps are as follows:

Standardized Aneurysm Clipping Procedure

A craniotomy is performed through a frontotemporal approach, including the pterional and supraorbital lateral routes. The aneurysm is exposed under a microscope, with full exposure of the neck of the aneurysm and careful dissection of surrounding important structures. Afterward, an aneurysm clip is used to occlude the aneurysm.

Ventricular Intracranial Pressure Monitoring Procedure

In the experimental group, the ipsilateral frontal horn puncture site (Kocher’s point) is exposed at the surgical incision. After drilling a small hole in the skull, a ventricular ICP monitoring system is placed through the frontal horn of the ventricle. The initial ICP is recorded, the drainage tube is clipped off, and the ventricular drainage tube is tunneled subcutaneously for more than 5 cm. Subsequently, conventional craniotomy and aneurysm clipping are performed through the same incision. During the surgery, approximately 20 ml of cerebrospinal fluid is slowly released, ICP data and intraoperative complications are recorded, and postoperatively, cerebrospinal fluid is continuously drained while ICP is monitored and regulated in real-time.

Control Group

Prior to the aneurysm clipping surgery, a lumbar cistern shunt is placed. Approximately 20 ml of cerebrospinal fluid is slowly released through the lumbar cistern drainage tube before the aneurysm clipping procedure. Prior to skull closure, a parenchymal-type ICP monitoring system is placed.

Postoperative Placement of Ventricular and Lumbar Cistern Drainage Tubes

The drainage tubes are kept in place for five to seven days. The drainage bag is hung at the head of the bed, with the drainage tube positioned approximately 15 cm above the plane of the lateral ventricle. Routine cerebrospinal fluid drainage is controlled at a rate of 100 ml per day, as indicated in the medical orders.

Postoperative Intracranial Pressure Measurement

Postoperatively, ICP was measured using either a ventricular or parenchymal ICP monitoring device. The target ICP was controlled to not exceed 20 mmHg (ICP-guided management). If ICP increased above 20 mmHg, intracranial pressure reduction interventions were completed within 15 minutes.

Data collection and evaluation metrics

Preoperative data included age, sex, comorbidities (hypertension, diabetes), aneurysm size, preoperative Hunt-Hess score, and modified Fisher grade; intraoperative data included operation time, intraoperative blood loss, the occurrence of malignant brain edema, and brain tissue damage; postoperative data included complications such as cerebral infarction, pulmonary infection, urinary tract infection, hydrocephalus, seizures, intracranial infection, number of lumbar punctures, average ICP changes, 72-hour postoperative brain edema (simplified early brain edema score (SEBES) score), length of hospital stay, and Glasgow Outcome Scale (GOS) score at discharge. Diagnosis of cerebral infarction was based on the appearance of new low-density areas on CT imaging. The degree of brain edema was assessed using the early brain edema score (SEBES) for aSAH, with mild edema (SEBES 0-2) and severe edema (SEBES 3-4). Postoperative neurological recovery was assessed using the GOS, categorizing recovery as good (GOS 4-5) or poor (GOS 1-3).

Statistical analysis

Statistical analysis was performed using IBM SPSS Statistics for Windows, Version 26 (Released 2019; IBM Corp., Armonk, New York, United States). Continuous variables were expressed as mean ± standard deviation, with intergroup comparisons conducted using independent samples t-test. Categorical variables were analyzed using the chi-square test. A p-value of <0.05 was considered statistically significant.

## Results

Comparison of clinical data between the two groups

There were no significant differences between the experimental group (n=32) and the control group (n=35) in terms of age, sex, comorbidities, preoperative Hunt-Hess score, modified Fisher grade, and aneurysm size (P > 0.05, Table 1).

**Table 1:** Comparison of clinical data between the two groups

Clinical Data	Experimental Group (n=32)	Control Group (n=35)	χ²/t-value	P-value
Age (mean ± SD)	53.72 ± 7.01	54.09 ± 8.10	0.197	0.845
Male (n%)	14 (43.75)	15 (42.86)	0.005	0.942
Comorbidities (n%)				
Hypertension	11 (34.38)	14 (40)	0.22	0.641
Diabetes	2 (6.25)	3 (8.57)	0.127	0.723
Hunt-Hess Grade (n%)			0.968	0.329
Grade I	0 (0.00）	3 (8.57）		
Grade Ⅱ	17 (53.12）	22 (62.85）		
Grade Ⅲ	9 (28.13）	3 (8.57）		
Grade Ⅳ	5 (15.63）	5 (14.28）		
Grade Ⅴ	1 (3.12）	2 (5.73）		
Fisher Grade (n%)			1.674	0.2
Grade 0	0 (0.00）	2 (5.72）		
Grade 1	4 (12.5）	1 (2.86）		
Grade 2	8 (25）	18 (51.42）		
Grade 3	3 (9.38）	0 (0.00）		
Grade 4	17 (53.12）	14 (40）		
Aneurysm Size (n%)			0.154	0.696
<5mm (Small）	11 (34.38)	12 (28.93)		
5-10mm (Medium）	20 (62.5)	20 (62.5)		
>10mm (Large）	1 (3.12)	3 (8.57)		

Intraoperative data

The average ICP during surgery in the experimental group was 16.4 ± 3.1 mmHg, with no instances of acute brain swelling. In contrast, the control group had two patients who experienced acute brain swelling and brain tissue damage. There were no significant differences between the two groups in terms of operation time and intraoperative blood loss (P > 0.05) (Table 2).

**Table 2:** Comparison of operation time and intraoperative blood loss between the two groups

Item	Experimental Group (n=32)	Control Group (n=35)	t-value	P-value
Operation time (h, mean ± SD)	4.78 ± 0.78	5.25 ± 1.22	1.86	0.067
Intraoperative blood loss (mL, mean ± SD)	375.00 ± 119.14	464.29 ± 313.56	1.51	0.135

Postoperative intracranial pressure changes

**Figure 1:**
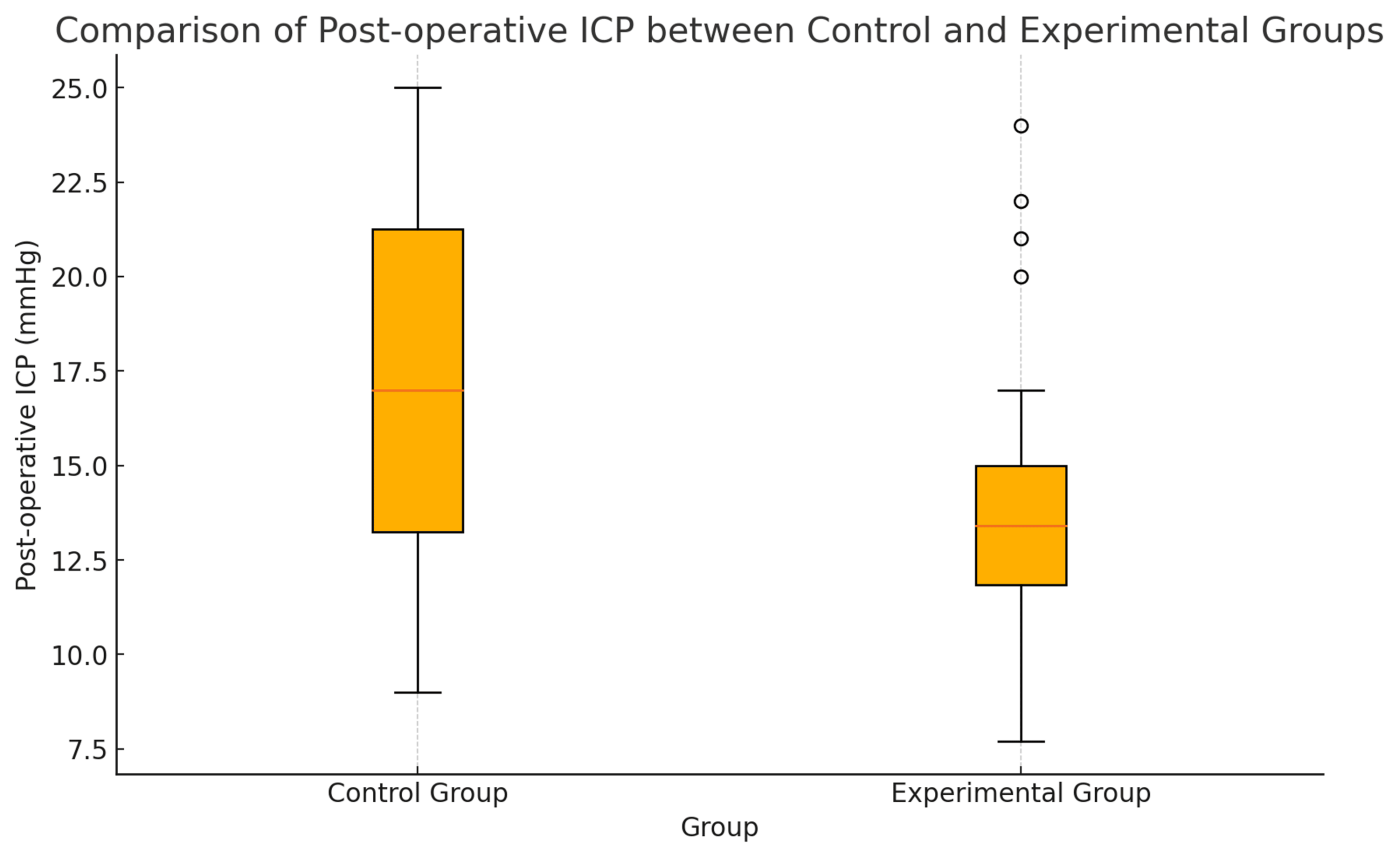
Comparison of postoperative ICP boxplot The experimental group showed a more concentrated range of postoperative ICP values, with fewer high-value outliers compared to the control group. In contrast, the control group exhibited a wider range of postoperative ICP values, with both the median and upper quartile values being higher than those in the experimental group. ICP: intracranial pressure

The average postoperative ICP in the experimental group was significantly lower than that in the control group (13.93 ± 3.76 mmHg vs. 17.08 ± 4.70 mmHg, P < 0.05) (Table 3). The experimental group exhibited a lower and more stable postoperative ICP (Figure 1), indicating that ventricular ICP monitoring has a significant advantage in controlling postoperative ICP.

**Table 3:** Comparison of postoperative ICP between the two groups of patients ICP: intracranial pressure

Item	Experimental Group (n=32)	Control Group (n=35)	t-value	P-value
Postoperative ICP (mmHg, mean ± SD)	13.93 ± 3.76	17.08 ± 4.70	3.04	0.003

Clinical outcomes

Postoperative Complications

The incidence of postoperative cerebral infarction was 9.38% in the experimental group and 14.29% in the control group, with no statistically significant difference (P > 0.05). The incidence of postoperative intracranial infections was 3.12% in the experimental group and 8.57% in the control group, also showing no statistically significant difference (P > 0.05). The number of lumbar punctures performed postoperatively showed no significant difference between the two groups (P > 0.05).

Regarding the SEBES score for cerebral edema at 72 hours postoperatively, the incidence of severe cerebral edema was lower in the experimental group compared to the control group, and this difference was statistically significant (P < 0.05). Other complications, such as pulmonary infection, urinary tract infection, hydrocephalus, and seizures, showed no statistically significant differences between the groups (P > 0.05) (Table 4).

**Table 4:** Comparison of postoperative complications between the two groups SEBES: simplified early brain edema score; GOS: Glasgow Outcome Scale

Complication	Experimental Group (n=32)	Control Group (n=35)	χ²/t-value	P-value
Intracranial infection (%)	1 (3.12)	3 (8.57)	0.868	0.355
Cerebral infarction (%)	3 (9.38)	5 (14.29)	0.374	0.543
Pulmonary infection (%)	15 (46.88)	22 (62.86)	1.72	0.194
Urinary tract infection (%)	5 (15.62)	4 (11.43)	0.247	0.621
Hydrocephalus (%)	1 (3.12)	2 (5.71)	0.255	0.615
Seizures (%)	1 (3.12)	2 (5.71)	0.255	0.615
Lumbar puncture (n%)	15 (46.88)	11（31.43）	0.374	0.543
SEBES score at 72 hours postoperatively			4.89	0.027
Severe edema (SEBES 3-4)	5 (15.62)	14 (40.00)		
Mild edema (SEBES 0-2)	27 (84.38)	21 (60.00)		
GOS score at discharge			4.92	0.027
Poor recovery (GOS 1-3)	3 (9.38)	11 (31.43)		
Good recovery (GOS 4-5)	29 (90.62)	24 (68.57)		

Length of Hospital Stay

The average length of stay was significantly shorter in the experimental group (18.03 ± 3.59 days) compared to the control group (20.49 ± 5.63 days, P < 0.05) (Table 5).

**Table 5:** Comparison of length of hospital stay and discharge GOS score between the two groups GOS: Glasgow Outcome Scale

Item	Experimental Group (n=32)	Control Group (n=35)	χ²/t-value	P-value
Length of stay (days, mean ± SD)	18.03 ± 3.59	20.49 ± 5.63	2.106	0.039
GOS score (n%)			5.285	0.007
1	0	0		
2	1 (3.12)	1 (2.86)		
3	2 (6.25)	10 (28.57)		
4	5 (15.63)	10 (28.57)		
5	24 (75.00)	14 (40.00)		

Postoperative Neurological Recovery

The Glasgow Outcome Scale (GOS) score at discharge indicated that the experimental group had a higher rate of good recovery (90.63%) compared to the control group (68.57%, P < 0.05) (Table 5).

## Discussion

This study explores the clinical application of ventricular ICP monitoring in anterior circulation ruptured aneurysm clipping surgery. In this study, we found that the use of this monitoring technology allows for real-time monitoring of ICP changes during and after the surgery. Draining cerebrospinal fluid reduces ICP, improves intraoperative and postoperative ICP control, reduces the occurrence of intraoperative brain tissue damage, prevents malignant brain tissue expansion, and lowers the incidence of postoperative complications.

Existing literature has reported that ICP monitoring plays an important role in the surgical management of severe traumatic brain injury and ruptured intracranial aneurysms [8]. Traditional ICP monitoring methods are usually independent of the surgical incision, requiring an additional incision. This not only increases the complexity of the procedure but also prolongs the surgery time and increases the risk of infection [9]. Preoperative placement of a lumbar cistern drainage tube increases surgical time and postoperative nursing difficulty [10]. Compared to the method based on ventricular ICP monitoring used by Luciano MG et al., this study avoids the trauma of an additional surgical incision and achieves precise ICP control [11]. This method improves the safety of the surgery and provides an important guarantee for postoperative ICP management. Compared to lumbar cistern drainage, the placement of a ventricular drainage tube did not increase the incidence of postoperative hydrocephalus, lumbar punctures, or intracranial infections. The incidence of postoperative intracranial infection was not significantly higher, possibly due to the more comprehensive surgical disinfection and draping using the same incision, as well as the standardized management of the cerebrospinal fluid drainage tube.

The effectiveness of ventricular ICP monitoring in ruptured aneurysm surgery is primarily attributed to its precise ICP monitoring capability [11]. Patients with ruptured aneurysms often present with brain edema and cerebral vasospasm, which can increase ICP both during and after surgery, thereby increasing the risk of ischemia and brain tissue damage [12]. By performing ventricular puncture and ICP monitoring through the same incision, the surgeon can monitor real-time changes in ICP during the procedure, allowing for timely adjustments to surgical techniques or pharmacological interventions. This helps avoid sudden increases in ICP that could damage brain tissue and reduce the occurrence of malignant brain expansion [13]. Postoperative ICP monitoring helps track the development of intracranial pressure, allowing for the early detection and management of potential complications, significantly reducing the risk of postoperative rebleeding and brain herniation. Compared to parenchymal ICP monitoring, ventricular ICP monitoring provided more concentrated data, with fewer high-value outliers. The ventricular ICP monitoring exhibited lower, more stable, and more accurate ICP levels [14]. This precise and stable monitoring ensures a smooth postoperative transition in ICP, providing favorable conditions for the patient’s recovery.

Compared to previous studies, this research clinically validates the value of ventricular ICP monitoring in ruptured aneurysm surgery through clinical data [15-17]. The innovation of this study lies in performing two key procedures through a single incision, effectively reducing the incidence of postoperative complications. Notably, the incidence of severe brain edema within 72 hours after surgery was lower, the rate of good neurological recovery postoperatively was higher, and the patient's hospital stay was shortened. This suggests that the introduction of ventricular ICP monitoring in aneurysm clipping surgeries not only enhances the safety of the procedure but also significantly improves postoperative outcomes for patients. This provides a new approach for the application of ICP monitoring in aneurysm surgeries and promotes the optimization of surgical techniques.

However, this study has certain limitations. First, as a retrospective study, there is a potential for selection bias. Although we made efforts to match the baseline characteristics of the two groups, there may still be uncontrolled confounding factors that affect the accuracy of the results. Second, the sample size of the study is relatively small. Although some results were not statistically significant, a larger-scale study could reveal more pronounced differences, such as the incidence of postoperative cerebral infarction. Additionally, for aneurysms located in different regions, ICP monitoring may introduce biases in the results. Finally, although the surgical procedures in this study were standardized, differences in the experience and technical skills of individual surgeons may have had an impact on the surgical outcomes.

Future studies should conduct larger-scale prospective randomized controlled trials to ensure the broad applicability of the research findings and to evaluate the long-term outcomes of patients who undergo aneurysm surgery with this technique. Additionally, long-term follow-up studies will help assess the impact of this technology on patients' long-term prognosis, including key indicators such as postoperative quality of life and neurological recovery.

## Conclusions

This study aims to evaluate the effectiveness of ventricular ICP monitoring in the treatment of anterior circulation ruptured aneurysm clipping. Through ventricular ICP monitoring, ICP can be effectively controlled after aneurysm clipping surgery, reducing postoperative complications, shortening hospital stays, and improving neurological recovery in patients. Therefore, this study finds that ventricular ICP monitoring provides a safer and more effective ICP monitoring solution for the treatment of anterior circulation ruptured aneurysms.
